# Differential hepatotoxicity of dietary and DNL-derived palmitate in the methionine-choline-deficient model of steatohepatitis

**DOI:** 10.1186/s12876-015-0298-y

**Published:** 2015-06-24

**Authors:** Andrew A. Pierce, Michael K. Pickens, Kevin Siao, James P. Grenert, Jacquelyn J. Maher

**Affiliations:** 1 Liver Center Laboratory, San Francisco General Hospital, University of California San Francisco, 1001 Potrero Avenue, Building 40, Room 4102, 94110 San Francisco, CA USA; 2 Department of Medicine, University of California San Francisco, San Francisco, USA; 3 Department of Pediatrics, University of California San Francisco, San Francisco, USA; 4 Department of Pathology, University of California San Francisco, San Francisco, USA; 5 Present address: Mary Bridge Children’s Health Center, 311 S. L Street, 98405 Tacoma, WA USA

**Keywords:** Liver, Fatty liver, Lipotoxicity, Saturated fat, De novo lipogenesis, Macronutrient

## Abstract

**Background:**

Saturated fatty acids are toxic to liver cells and are believed to play a central role in the pathogenesis of non-alcoholic steatohepatitis. In experimental steatohepatitis induced by feeding mice a methionine-choline-deficient (MCD) diet, the degree of liver damage is related to dietary sugar content, which drives de novo lipogenesis and promotes the hepatic accumulation of saturated fatty acids. The objective of this study was to determine whether dietary palmitate exerts the same toxicity as carbohydrate-derived palmitate in the MCD model of fatty liver disease.

**Methods:**

We fed mice custom MCS and MCD formulas containing 4 different carbohydrate-fat combinations: starch-oleate, starch-palmitate, sucrose-oleate and sucrose-palmitate.  After 3 wk, we compared their metabolic and disease outcomes.

**Results:**

Mice fed the custom MCD formulas developed varying degrees of hepatic steatosis and steatohepatitis, in the order starch-oleate < starch-palmitate < sucrose-oleate < sucrose-palmitate. Liver injury correlated positively with the degree of hepatic lipid accumulation. Liver injury also correlated positively with the amount of palmitate in the liver, but the relationship was weak. Importantly, mice fed MCD starch-palmitate accumulated as much hepatic palmitate as mice fed MCD sucrose-oleate, yet their degree of liver injury was much lower. By contrast, mice fed MCD sucrose-palmitate developed severe liver injury, worse than that predicted by an additive influence of the two nutrients.

**Conclusion:**

In the MCD model of steatohepatitis, carbohydrate-derived palmitate in the liver is more hepatotoxic than dietary palmitate. Dietary palmitate becomes toxic when combined with dietary sugar in the MCD model, presumably by enhancing hepatic de novo lipogenesis.

## Background

Saturated fatty acids (SFA) are important mediators of hepatic lipotoxicity [1–5] and have been implicated in the pathogenesis of non-alcoholic steatohepatitis (NASH). This is particularly true in the case of experimental NASH induced by a methionine-choline-deficient (MCD) diet [3, 6]. MCD feeding induces at least two major alterations in hepatic lipid metabolism that contribute to SFA accumulation in the liver: it impairs hepatic lipid export by interfering with VLDL synthesis [6, 7], and suppresses stearoyl-CoA desaturase-1 (SCD1) through an as-yet unidentified mechanism [8]. SFA accumulation in the livers of MCD-fed mice and the accompanying liver injury can be modulated by altering the carbohydrate composition of the MCD formula. Our laboratory has shown that enriching the diet with simple sugar enhances steatohepatitis, whereas substituting dietary sugar with complex carbohydrate reduces liver injury [6, 9]. These studies indicate that sucrose-stimulated de novo lipogenesis (DNL) is an important prerequisite to liver pathology in the MCD model. Specifically, they implicate palmitate (C16:0), the product of DNL, as a mediator of steatohepatitis in vivo.

It is known that hepatic fatty acids derive from three sources: dietary fat, hepatic DNL and adipose tissue lipolysis. Having demonstrated that DNL-derived palmitate is injurious to the liver of MCD-fed mice, we questioned whether palmitate within dietary fat is similarly hepatotoxic. Evidence indicates that different types of fatty acids (saturated, unsaturated, polyunsaturated) undergo different metabolic fates in animals and humans [10–14], but it is unknown whether the same fatty acid always behaves identically regardless of its origin (diet or DNL). The objective of this study was to compare the hepatotoxicity of MCD formulas in which hepatic palmitate derives primarily from DNL, primarily from the diet, or both.

## Methods

### Dietary studies

Adult male C3H/HeOuJ mice (The Jackson Laboratory, Bar Harbor, ME) were fed for 21 days ad libitum with one of 8 custom methionine-choline-sufficient (MCS) or MCD formulas (Dyets, Inc., Bethlehem, PA). Each formula contained a unique combination of carbohydrate and fat as detailed in Table 1. The formulas were named for their primary carbohydrates and fats: starch-oleate, starch-palmitate, sucrose-oleate and sucrose-palmitate. All formulas contained 18 % protein, 64 % carbohydrate and 10 % fat by weight. Paired MCS and MCD formulas were matched for all nutrients except L-methionine and choline chloride. At the end of the study period, mice were fasted for 4 h prior to killing. Serum alanine aminotransferase (ALT) was measured on an ADVIA 1800 autoanalyzer (Siemens Healthcare Diagnostics, Deerfield, IL).

**Table 1:** Composition of custom MCS and MCD formulas

	MCS				MCD			
	Starch-oleate	Starch-palmitate	Sucrose-oleate	Sucrose-palmitate	Starch-oleate	Starch-palmitate	Sucrose-oleate	Sucrose-palmitate
Protein (g/kg)								
L-arginine (free base)	6.3	6.3	6.3	6.3	6.3	6.3	6.3	6.3
L-histidine (free base)	4.5	4.5	4.5	4.5	4.5	4.5	4.5	4.5
L-lysine	16.1	16.1	16.1	16.1	16.1	16.1	16.1	16.1
L-tyrosine	9.2	9.2	9.2	9.2	9.2	9.2	9.2	9.2
L-tryptophan	2.1	2.1	2.1	2.1	2.1	2.1	2.1	2.1
L-phenylalanine	8.7	8.7	8.7	8.7	8.7	8.7	8.7	8.7
L-cysteine	3.7	3.7	3.7	3.7	3.7	3.7	3.7	3.7
L-threonine	6.6	6.6	6.6	6.6	6.6	6.6	6.6	6.6
L-leucine	15.3	15.3	15.3	15.3	15.3	15.3	15.3	15.3
L-isoleucine	8.4	8.4	8.4	8.4	8.4	8.4	8.4	8.4
L-valine	9.9	9.9	9.9	9.9	9.9	9.9	9.9	9.9
Glycine	3.1	3.1	3.1	3.1	3.1	3.1	3.1	3.1
L-proline	20.4	20.4	20.4	20.4	20.4	20.4	20.4	20.4
L-glutamic acid	36.2	36.2	36.2	36.2	36.2	36.2	36.2	36.2
L-alanine	4.5	4.5	4.5	4.5	4.5	4.5	4.5	4.5
L-aspartic acid	11.3	11.3	11.3	11.3	11.3	11.3	11.3	11.3
L-serine	9.4	9.4	9.4	9.4	9.4	9.4	9.4	9.4
Carbohydrate (g/kg)								
Cornstarch	587.9	587.9	0.0	0.0	591.9	591.9	0.0	0.0
Dyetrose	50.0	50.0	50.0	50.0	50.0	50.0	50.0	50.0
Sucrose	0.0	0.0	587.9	587.9	0.0	0.0	591.9	591.9
Cellulose	30.0	30.0	30.0	30.0	30.0	30.0	30.0	30.0
Fat (g/kg)								
Tripalmitin	0.0	100.0	0.0	100.0	0.0	100.0	0.0	100.0
High-oleate (85 %) sunflower oil	100.0	0.0	100.0	0.0	100.0	0.0	100.0	0.0
Methionine and choline (g/kg)								
L-methionine	2.0	2.0	2.0	2.0	0.0	0.0	0.0	0.0
Choline chloride	2.0	2.0	2.0	2.0	0.0	0.0	0.0	0.0
Additives (g/kg)								
Salt mix #210030	35.0	35.0	35.0	35.0	35.0	35.0	35.0	35.0
Sodium bicarbonate	7.4	7.4	7.4	7.4	7.4	7.4	7.4	7.4
Vitamin Mix #310025	10.0	10.0	10.0	10.0	10.0	10.0	10.0	10.0
Total (g/kg)	1000.0	1000.0	1000.0	1000.0	1000.0	1000.0	1000.0	1000.0
Protein	18 %	18 %	18 %	18 %	18 %	18 %	18 %	18 %
CHO	64 %	64 %	64 %	64 %	64 %	64 %	64 %	64 %
Fat	10 %	10 %	10 %	10 %	10 %	10 %	10 %	10 %
Fiber	3 %	3 %	3 %	3 %	3 %	3 %	3 %	3 %

All animals received humane care according to guidelines published by the US Public Health Service. All experimental procedures were approved by the Institutional Animal Care and Use Committee at the University of California, San Francisco.

### Triglyceride and fatty acid analysis

Lipids were extracted from fresh liver tissue using the Folch method [15]. Aliquots were dried and resuspended in 1-butanol containing 0.01 % butyrated hydroxytoluene for measurement of total triglyceride (TR0100; Sigma Chemical Co., St. Louis, MO). Fatty acid analysis was performed on flash-frozen liver tissue. Lipid extraction and TrueMass® neutral lipid analysis were performed by Lipomics Technologies (West Sacramento, CA). Tissue samples were subjected to a combination of liquid- and solid-phase extraction procedures to separate neutral lipids from phospholipids, followed by thin-layer chromatography to separate neutral lipid classes and gas chromatography to quantitate individual fatty acids. All samples were processed in the presence of internal standards to monitor extraction efficiency and verify measurement accuracy.

### Evaluation of gene expression

Total RNA was extracted from liver using TRIzol reagent (Life Technologies, Carlsbad, CA) and purified using the RNeasy kit (Qiagen, Valencia, CA). RNA integrity was verified by formaldehyde gel electrophoresis. cDNA was synthesized using iScript (BioRad, Hercules, CA); quantitative PCR was performed with TaqMan® assay kits (Life Technologies, Carlsbad, CA) using β-glucuronidase as the internal control gene.

### Histologic analyses

Formalin-fixed, paraffin-embedded sections of liver tissue were stained with hematoxylin and eosin for routine histology. Apoptotic cells were identified in liver sections by terminal deoxynucleotide transferase-mediated deoxyuridine triphosphate nick end-labeling (TUNEL) (ApopTag Plus Peroxidase *In Situ* Apoptosis Detection Kit, Millipore, Billerica, MA). To assess hepatic inflammation, liver sections were stained with anti-CD11b (Abcam, Cambridge, MA). Collagen deposition was assessed by Sirius Red staining. Counting of TUNEL-positive or CD11b-positive cells was performed manually in 10 microscopic fields per liver, each measuring 0.4 mm^2^. Data were reported as the average number of cells per microscopic field. Sirius red-stained area was assessed by morphometry (Simple PCI, Hamamatsu Corporation, Sewickley, PA).

### Statistical methods

Experiments included 10 mice per diet group, performed in 2 separate cohorts of 5. Some outcome measures were assessed in only one cohort as described in the figure legends. Results were compared by analysis of variance with Tukey post-hoc testing. *P* values < 0.05 were considered statistically significant.

## Results and discussion

**Fig. 1:**
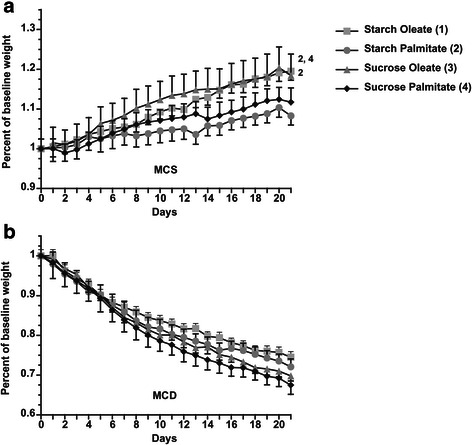
Weight gain/loss on MCS and MCD diets. **a** 21-day weight curve for mice fed MCS formulas. **b** 21-day weight curve for mice fed MCD formulas. Values represent mean ± SE for n = 10. Superscripts indicate *P* < 0.05 vs. comparison groups by number

Mice were fed custom MCS and MCD diets that differed from commercial MCS and MCD formulas by being nearly completely enriched with a single type of carbohydrate (sucrose or starch) or fat (palmitate or oleate). The custom MCS and MCD mixtures were designed to maximize palmitate accumulation in the liver via DNL (with sucrose) or diet (with palmitate) or both. Starch served as the control to sucrose, whereas oleate served as the control to palmitate. Mice in all 8 dietary groups ate comparable amounts of food during the study period. Animals fed MCS formulas gained weight (14.7 ± 1.3 %), whereas those fed MCD formulas lost weight (28.8 ± 1.0 %), which is characteristic for the dietary model [8]. All MCD-fed mice lost comparable amounts of weight regardless of the macronutrient composition of the diet (Fig. 1b). MCD feeding is unique in that it does not induce insulin resistance or hyperglycemia coincident with steatohepatitis [16]. This pattern did not change with the 4 custom MCD diets; there was no evidence of insulin resistance or hyperglycemia in any MCD group (not shown).

**Fig. 2:**
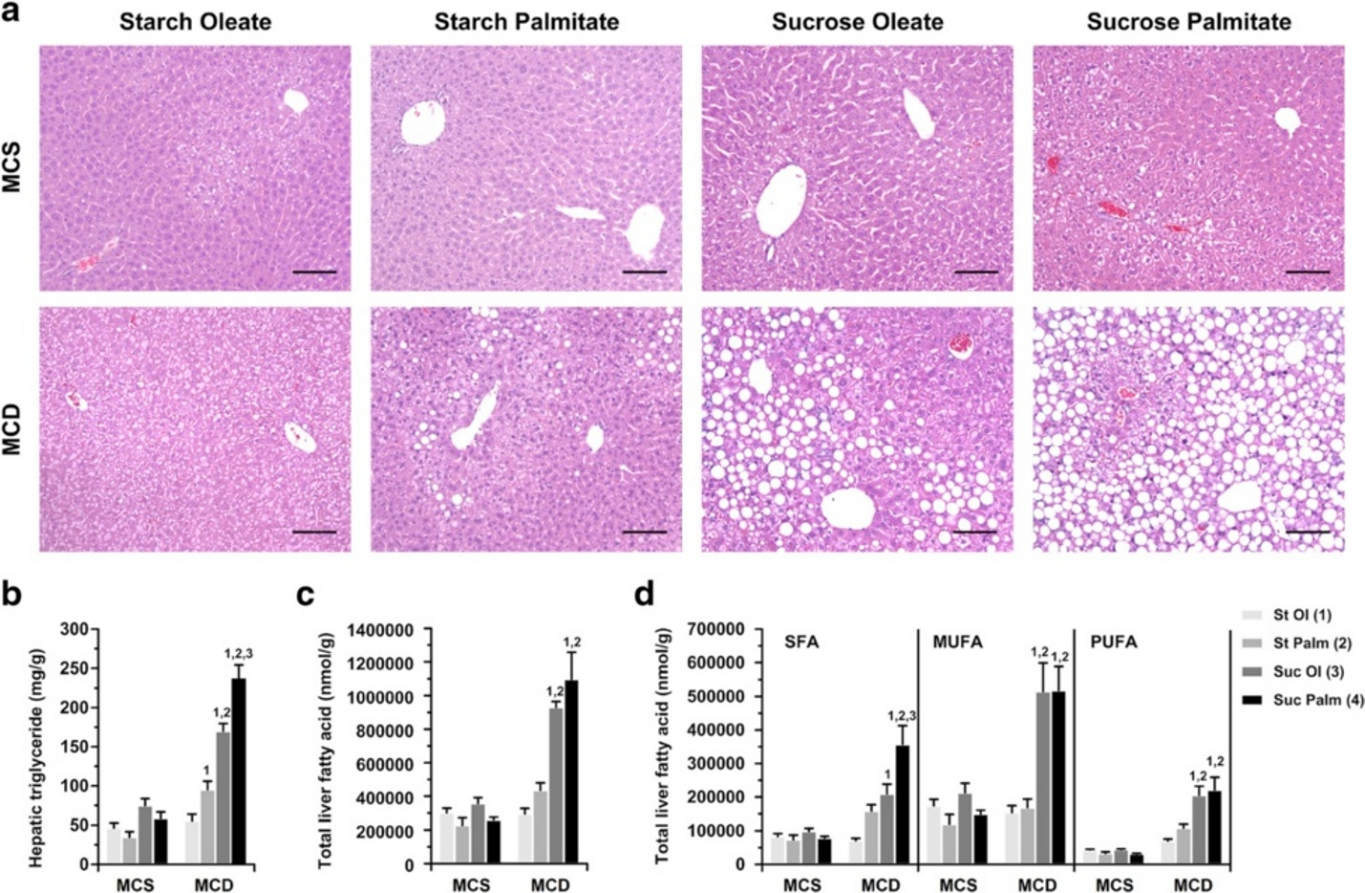
Hepatic lipid accumulation in mice fed custom MCS and MCD diets. **a** Photomicrographs illustrate liver histology after 21 days of MCS or MCD feeding. There was no apparent steatosis in any of the MCS-fed groups. MCD diets induced histologic steatosis of varying degrees depending upon macronutrient composition. Bar = 100 μm. **b** Total hepatic triglyceride measured biochemically in MCS and MCD livers at 21 days. Values represent mean ± SE for n = 10. **c** Total hepatic fatty acid content measured by gas chromatography and **d** total hepatic fatty acid segregated by SFA, MUFA and PUFA. Values represent mean ± SE for n = 5. St Ol = Starch Oleate, St Palm = Starch Palmitate, Suc Ol = Sucrose Oleate, Suc Palm = Sucrose Palmitate. Superscripts indicate *P* < 0.05 vs. MCD comparison groups by number

After 3 weeks on the custom diets, MCS-fed mice remained free of histologic hepatic steatosis. By contrast, MCD-fed mice developed markedly different degrees of hepatic steatosis depending on macronutrient composition. This was evident histologically (Fig. 2a) and confirmed by hepatic lipid quantitation [6, 9] (Fig. 2b and c). MCD formulas containing sucrose induced the most pronounced hepatic steatosis regardless of the accompanying type of dietary fat. The worst steatosis occurred in mice fed MCD diets containing both sucrose and palmitate.

**Fig. 3:**
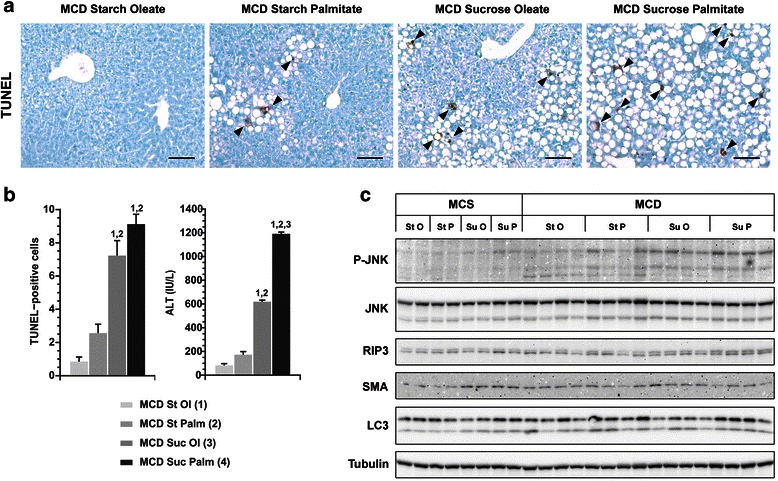
Liver injury in mice fed custom MCD diets. **a** Photomicrographs illustrate TUNEL staining in mice fed custom MCD formulas for 21 days. TUNEL-positive cells are marked with arrowheads. Bar = 100 μm. There were no TUNEL-stained cells in mice fed MCS formulas over this interval (not shown). **b** Graphs depict TUNEL- positive cells (average number of cells per 0.4 mm^2^ section) and serum ALT in MCD-fed livers. Values represent mean ± SE for n = 5 (TUNEL) and n = 10 (ALT). Superscripts indicate *P* < 0.05 vs. MCD comparison groups by number. **c** Western blots illustrate JNK phosphorylation and hepatic expression of of RIP3, smooth muscle actin (SMA) and LC3 in MCS- and MCD-fed mice. Tubulin is shown as a loading control. St O = Starch Oleate, St P Starch Palmitate, Suc O = Sucrose Oleate, Suc P = Sucrose Palmitate

Mice fed MCD formulas containing sucrose also exhibited the greatest degrees of liver injury, as shown by TUNEL staining and serum ALT (Fig. 3). Just as it induced the most steatosis, the MCD formula containing both sucrose and palmitate caused the worst hepatotoxicity. Accompanying the liver injury in MCD-fed mice was hepatic activation of Jun-N-terminal kinase (JNK); the greatest degree of JNK activation occurred in the sucrose-palmitate group. In addition to JNK, the necroptosis marker receptor-interacting protein kinase 3 (RIP3) was mildly upregulated in response to MCD feeding. RIP3 was most visible in mice fed sucrose-palmitate. LC3, a marker of autophagosomes, was up-regulated in mice fed MCD sucrose-palmitate, but also in mice fed MCD starch-palmitate. This suggests dietary fat is affecting hepatic autophagy either positively or negatively, but without a firm relationship to liver injury. Overall the data support the notion that dietary sucrose activates cytotoxicity pathways known to be operative in steatohepatitis (JNK, RIP3) [17, 18], and the addition of dietary palmitate accentuates these events.

**Fig. 4:**
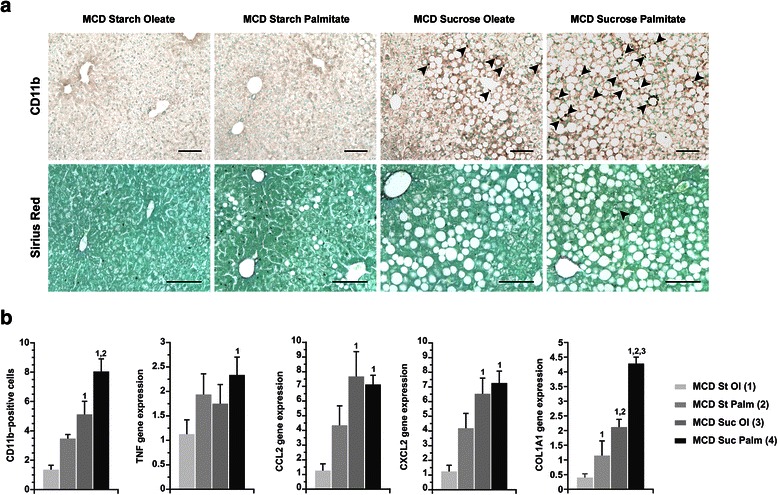
Hepatic inflammation and markers of fibrosis in mice fed custom MCD diets. **a** Photomicrographs illustrate infiltration of CD11b-positive leukocytes (arrowheads) and Sirius Red staining for connective tissue (arrowhead) in mice fed custom MCD formulas for 21 days. Bar = 100 μm. **b** Graphs depict CD11b-positive cells (average number of cells per 0.4 mm^2^ section) and relative hepatic expression of TNF, C-C chemokine ligand-2 (CCL2), CXC chemokine ligand-2 (CXCL2) and type I collagen (COL1A1). Values represent mean ± SE for n = 5. MCD St Ol = MCD Starch Oleate, MCD St Palm = MCD Starch Palmitate, MCD Suc Ol = MCD Sucrose Oleate, MCD Suc Palm = MCD Sucrose Palmitate. Superscripts indicate *P* < 0.05 vs. comparison groups by number

Hepatocellular injury in MCD-fed mice was accompanied by the induction of pro-inflammatory genes in the liver and the hepatic influx of CD11b-positive leukocytes (Fig. 4). The degree of hepatic inflammation mirrored the degree of hepatocellular injury in all MCD-fed groups. Stellate cell activation, characterized by the induction of type I collagen mRNA in the liver, was also affected by diet; again, MCD sucrose-palmitate provided the greatest stimulus to collagen gene regulation. Despite robust collagen gene induction in the livers of MCD-fed mice, there was no increase in smooth muscle-alpha-actin expression (Fig. 3c). Nor was there any evidence of collagen deposition in the liver by morphometry (<0.5 % Sirius Red-stained area in all groups). This suggests that collagen gene induction reflects acute liver injury rather than fibrosis at the 3-weeks time point, but portends fibrosis over a longer interval.

**Fig. 5:**
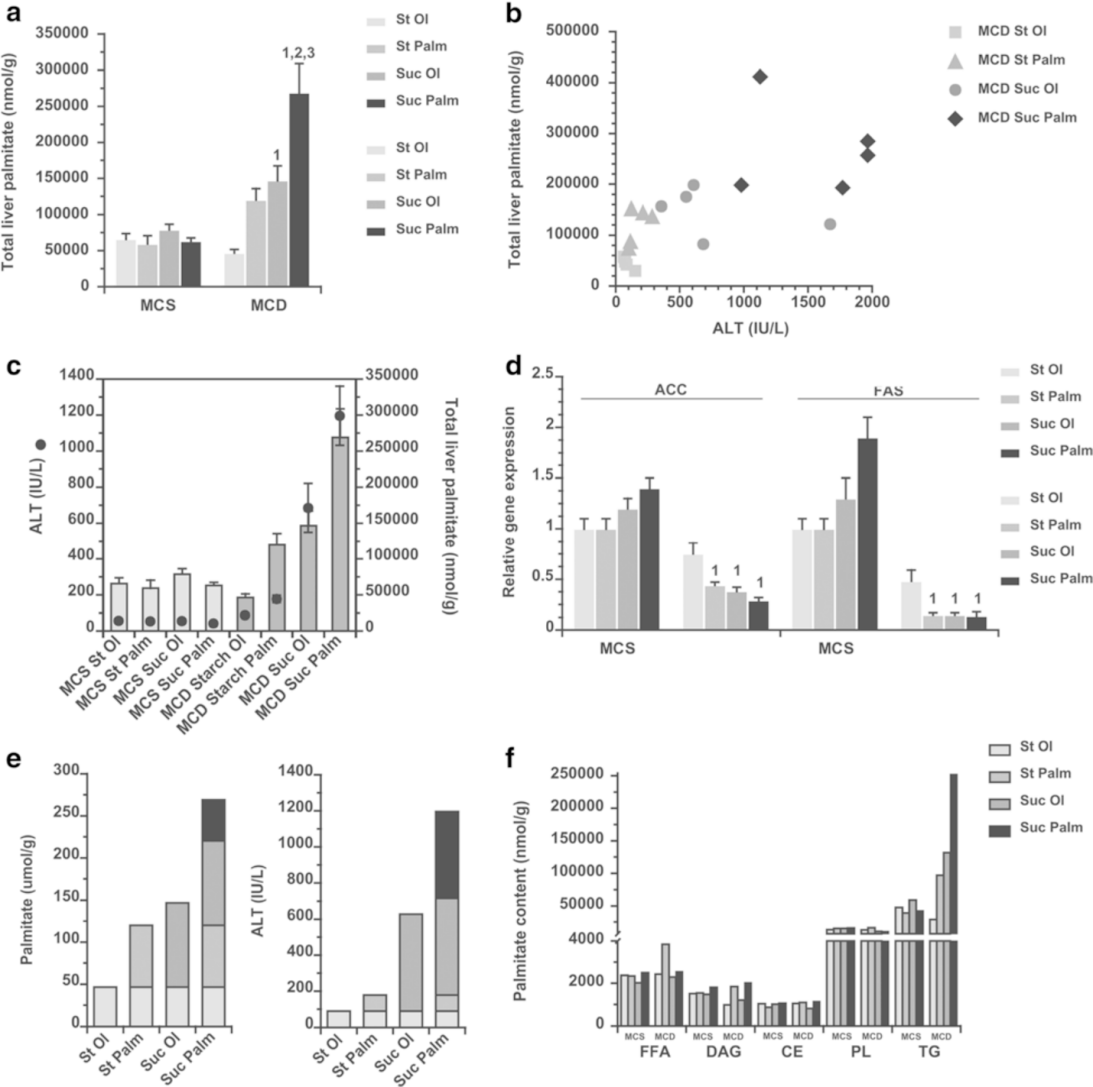
Impact of MCD diets on hepatic palmitate accumulation and relation to liver injury. **a** Hepatic palmitate levels in mice fed MCS or MCD diets for 21 days, measured by gas chromatography. Values represent mean ± SE for n = 5. St Ol = Starch Oleate, St Palm = Starch Palmitate, Suc Ol = Sucrose Oleate, Suc Palm = Sucrose Palmitate. * *P* < 0.05 vs. MCS formula of the same nutrient composition. Numerical superscripts indicate *P* < 0.05 vs. MCD comparison groups by number. **b** Scattergram demonstrating the relationship between total liver palmitate and serum ALT level in individual MCD-fed mice. **c** Graph showing the relationship between mean hepatic palmitate level and serum ALT in the 4 MCD-fed groups. Values represent mean ± SE for n = 10. **d** Hepatic expression of ACC and FAS mRNA in mice fed MCS or MCD diets for 21 days. Values represent mean ± SE for n = 5. Numerical superscripts indicate *P* < 0.05 vs. MCD comparison groups by number. **e** Left graph represents total hepatic palmitate as an estimate of the amount of excess palmitate attributable to the addition of palmitate, sucrose, or both to the MCD formula. Black segment demonstrates the amount of palmitate present in the liver that was not predicted by a simple additive effect of palmitate and sucrose. Right graph similarly represents serum ALT as an estimate of the amount of excess ALT attributable to the addition of palmitate, sucrose, or both to the MCD formula. Black segment shows the amount of ALT that was not predicted by an additive effect of palmitate and sucrose. **f** Graph depicts total liver palmitate within individual lipid compartments of MCS and MCD-fed livers: free fatty acids (FFA), diacylglycerols (DAG), cholesteryl esters (CE), phospholipids (PL) and triglycerides (TG)

After characterizing the effects of the 4 custom MCD formulas on liver injury, we explored whether the different outcomes of the 4 diets could be attributed to differences in hepatic palmitate accumulation. Hepatic palmitate levels rose above control values in MCD-fed mice whose diets contained sucrose or palmitate or both (Fig. 5a). Although palmitate levels tended to correlate positively with ALT levels in these MCD groups, the relationship was not strong (Fig. 5b). Indeed, as shown in Fig. 5c, mice fed MCD sucrose-oleate accumulated no more palmitate than those fed MCD starch-palmitate, yet their ALT levels were significantly higher. This suggests that palmitate arising from sucrose in the diet (DNL palmitate) is more hepatotoxic than palmitate coming directly from the diet in the MCD model of liver disease. We assessed lipogenic gene expression in all mice, although previous studies have shown gene expression does not correlate with actual DNL in the MCD model of steatohepatitis [6, 8]. MCD-fed mice displayed marked suppression of mRNA encoding acetyl-CoA carboxylase (ACC) and fatty acid synthase (FAS) compared to MCS controls, as has been reported previously [8] (Fig. 5d). There were no differences in lipogenic gene expression among the 3 custom MCD groups that accumulated hepatic palmitate, but the predictive value of this observation is low.

Noteworthy was that mice fed the MCD formula containing both sucrose and palmitate accumulated more hepatic palmitate and had higher ALT levels than would have been predicted by a mere additive effect of both nutrients (Fig. 5e). Dietary saturated fat is known to stimulate hepatic DNL [19], and thus the excess palmitate in the livers of mice fed MCD sucrose-palmitate likely derives from exaggerated DNL. The extremely high ALT levels in these mice underscores that palmitate arising from DNL is particularly noxious to the liver.

Overall, the current experiments confirm our previous observation that dietary sucrose, through DNL conversion to palmitate in the liver, is an important inducer of liver injury when downstream pathways for fatty acid desaturation and lipid excretion are blocked [6]. More importantly, they extend previous work by demonstrating that dietary palmitate does not induce the same level of hepatotoxicity as DNL palmitate despite accruing to twice the concentration found in control livers (Fig. 5c). This suggests that dietary palmitate is handled differently by the liver than DNL palmitate. Different metabolic fates for DNL vs. exogenous palmitate have been reported in cultured adipocytes and HepG2 cells [20, 21]. The noted differences, however, were in palmitate desaturation and elongation, which in MCD livers would not likely affect lipotoxicity. We searched individual hepatic compartments in MCD-fed mice to determine whether palmitate accumulates preferentially in the more metabolic depots such as free fatty acids or diacylglycerols, but found excess palmitate only in hepatic triglycerides (Fig. 5f). It is possible that liver injury is a function of the lability of the hepatic triglyceride pool in these mice; we could not determine this in the current experiments.

The fact that MCD starch-palmitate mice were relatively free of liver injury, whereas MCD sucrose-palmitate mice had exaggerated liver injury, supports the concept that dietary saturated fat by itself is nearly innocuous to the liver but becomes toxic only in combination with dietary sugar. This is an intriguing theory, but unfortunately, saturated fat consumption is unlikely to be uncoupled from sugar consumption in free-living humans. Our other major finding, that sucrose and palmitate together induce synergistic hepatotoxicity in mice, is more translationally relevant. Indeed, dietary saturated fat has recently been shown to enhance hepatic steatosis, if not steatohepatitis, in humans when added to a mixed-nutrient diet [22]. Given our current experimental results, it will be important to determine whether synergy between sucrose and palmitate is an important inducer of liver injury when taken out of the context of the MCD model. Such experiments are currently underway.

## Conclusion

In summary, this study demonstrates that saturated fatty acids produced in the liver via DNL are more hepatotoxic than those reaching the liver directly from the diet.  Our findings in mice parallel observations in humans that DNL is an important contributor to fatty liver disease, and suggest that in humans as well, sugar consumption may be more harmful to the liver than consumption of saturated fat. Importantly, our findings indicate that a diet containing both sugar and saturated fat is more harmful to the liver than a diet containing either nutrient alone. This is likely related to synergy between sugar and saturated fat in stmulating DNL.

## Abbreviations

ACC: Acetyl-CoA carboxylase
ALT: Alanine aminotransferase
CCL2: C-C chemokine ligand-2
COL1A1: Collagen type Ia1
CE: Cholesteryl ester
CXCL2: CXC chemokine ligand-2
DAG: Diacylglycerol
DNL: De novo lipogenesis
FAS: Fatty acid synthase
FFA: Free fatty acid
JNK: Jun N-terminal kinase
MCD: Methionine-choline-deficient
MCS: Methionine-choline-sufficient
MUFA: Monounsaturated fatty acid
NASH: Non-alcoholic steatohepatitis
PUFA: Polyunsaturated fatty acid
PL: Phospholipid
RIP3: Receptor-interacting protein kinase 3
SCD1: Stearoyl-CoA desaturase-1
SFA: Saturated fatty acid
SMA: Smooth muscle actin
TG: Triglyceride
TUNEL: Terminal deoxynucleotide transferase-mediated deoxyuridine triphosphate nick end-labeling
